# A policy assessment tool to identify causes of inequities that influence obesity prevalence

**DOI:** 10.2471/BLT.24.292061

**Published:** 2024-12-03

**Authors:** Tim Lobstein, Mojca Gabrijelčič

**Affiliations:** a World Obesity Federation, 3 Waterhouse Square, London, EC1N 2SW, England.; b National Institute of Public Health, Ljubljana, Slovenia.

## Abstract

When policy-makers propose health-related initiatives they need to assess the impact on health inequalities, including disparities in diet-related diseases and obesity. Health impact assessments, including health equity assessments, can provide insights into the potential health outcomes, but they are usually based on engagement with stakeholders and beneficiaries and their quality is not easy to evaluate. In this paper, we propose a policy assessment tool designed to ask a set of questions on the impact on health equity of policies and interventions that may be answerable from empirical evidence or from public health principles. The results can be graded by strength of the impact and direction of the likely effects, and can provide a summary of how a policy or initiative may increase or decrease health inequity. The questions can be used as part of the scoping review for an impact assessment. We describe the application of this approach to the evaluation of three population-based policies to assess their likely impact on obesity inequalities: (i) policies to restrict children’s exposure to the promotional marketing of unhealthy foods and beverages; (ii) policies on food and beverage reformulation; and (iii) policies to improve food procurement for public institutions.

## Résumé

Lorsque les décideurs politiques proposent des initiatives dans le domaine de la santé, ils doivent en évaluer l’impact sur les inégalités en matière de santé, y compris sur les disparités dans les maladies liées aux régimes alimentaires et à l’obésité. Les évaluations de l’impact sur la santé, y compris l’évaluation de l’équité en matière de santé, permettent de fournir des indications sur les résultats potentiels pour la santé, mais elles sont généralement basées sur l’engagement avec les parties prenantes et les bénéficiaires, et leur qualité n’est pas facile à évaluer. Le présent article propose un outil d’évaluation des politiques conçu pour poser une série de questions sur l’impact des politiques et des interventions sur l’équité en matière de santé, questions auxquelles il est possible de répondre à partir de données empiriques ou de principes de santé publique. Les résultats peuvent être classés selon la force de l’impact et la direction des effets probables. Ils sont également de nature à fournir un résumé de la manière dont une politique ou une initiative est susceptible d’accroître ou de réduire les inégalités en matière de santé. Les questions peuvent servir dans le cadre de l’examen de la portée d’une étude d’impact. Nous décrivons l’application de cette approche à l’évaluation de trois politiques fondées sur les populations afin d’évaluer leur impact probable sur les inégalités en matière d’obésité: (i) des politiques visant à limiter l’exposition des enfants au marketing promotionnel d’aliments et de boissons malsains; (ii) des politiques sur la reformulation des aliments et des boissons; et (iii) des politiques visant à améliorer l’approvisionnement alimentaire des établissements publics.

## Resumen

Cuando los responsables de elaborar las políticas proponen iniciativas relacionadas con la salud, deben evaluar su impacto en las desigualdades sanitarias, incluidas las diferencias en las enfermedades relacionadas con la dieta y la obesidad. Las evaluaciones del impacto sanitario, incluida la evaluación de la equidad en salud, pueden aportar información sobre los posibles resultados sanitarios, pero suelen apoyarse en la participación de las partes interesadas y los beneficiarios, y su calidad no es fácil de evaluar. En este documento, se propone una herramienta de evaluación de políticas diseñada para plantear una serie de preguntas sobre el impacto en la equidad sanitaria de políticas e intervenciones que puedan responderse a partir de pruebas empíricas o de principios de salud pública. Los resultados pueden calificarse según la fuerza del impacto y la dirección de los efectos probables, y pueden proporcionar un resumen de cómo una política o iniciativa puede aumentar o disminuir la desigualdad sanitaria. Las preguntas pueden utilizarse como parte del examen del alcance de una evaluación de impacto. Se describe la aplicación de este enfoque a la evaluación de tres políticas basadas en la población para valorar su posible impacto en las desigualdades en materia de obesidad: (I) políticas para restringir la exposición de los niños al marketing promocional de alimentos y bebidas poco saludables; (ii) políticas sobre reformulación de alimentos y bebidas; y (iii) políticas para mejorar la adquisición de alimentos para instituciones públicas.

## ملخص

عندما يقترح صناع السياسات مبادرات تتعلق بالصحة، فإنهم يحتاجون إلى تقييم التأثير على حالات عدم المساواة في الصحة، بما في ذلك الفروقات المرتبطة بالنظام الغذائي والسمنة. إن تقييمات التأثير على الصحة، بما في ذلك تقييم المساواة في الصحة، يمكنها أن توفر رؤى حول النتائج الصحية المحتملة، ولكنها تستند عادةً إلى المشاركة مع أصحاب المصلحة والمستفيدين، وليس من السهل تقييم جودتها. نحن نقترح في هذه الورقة أداة لتقييم السياسات، مصممة لطرح مجموعة من الأسئلة حول تأثير السياسات والتدخلات على المساواة في الصحة، والتي قد تكون قابلة للإجابة من الأدلة التجريبية، أو من مبادئ الصحة العامة. يمكن تصنيف النتائج حسب قوة التأثير واتجاه التأثيرات المحتملة، ويمكن أن توفر ملخصًا لكيف يمكن لسياسة أو مبادرة زيادة عدم المساواة في الصحة، أو تقليلها. يمكن استخدام الأسئلة كجزء من مراجعة عن كثب لتقييم التأثير. نحن نصف تطبيق هذا الأسلوب على تقييم ثلاث سياسات قائمة على السكان لتقييم تأثيرها المحتمل على عدم المساواة في السمنة: (أ) سياسات تقييد تعرض الأطفال للتسويق الترويجي للأطعمة والمشروبات غير الصحية؛ و(ب) السياسات المتعلقة بإعادة صياغة الأطعمة والمشروبات؛ و(ج) سياسات تحسين شراء الأطعمة للمؤسسات العامة.

## 摘要

政策制定者在提出与健康相关的举措时，需要评估举措对健康不平等现象的影响，包括在饮食相关疾病和肥胖方面的差异。健康影响评估，包括健康公平评估，有助于深入了解潜在的健康结果，但这些评估通常基于利益攸关方和受益人的参与情况，其质量难以衡量。在本文中，我们提出了一种政策评估工具，以便就政策和干预措施对健康公平的影响提出一系列问题，并基于经验证据或公共卫生原则进行解答。评估结果可按照影响强度和可能造成影响的方面进行分级，并概述一项政策或举措会如何增加或减少健康不平等现象。这些问题可用作对影响评估结果进行范围综述的一部分。我们阐述了该方法在评估三项基于人群的政策中的应用情况，以评估这些政策可能对肥胖不平等现象造成的影响：(i) 限制儿童接触不健康食品和饮料相关市场推广活动的政策；(ii) 关于食品和饮料配方改革的政策；以及 (iii) 改善公共机构食品采购状况的政策。

## Резюме

Когда ответственные лица предлагают инициативы в области здравоохранения, им необходимо оценить их влияние на неравенство в сфере здравоохранения, включая неравенство в области заболеваний, связанных с питанием, и ожирения. Оценки воздействия на здоровье, включая оценку справедливости в отношении здоровья, могут дать представление о потенциальных результатах в сфере здравоохранения, однако они обычно основаны на взаимодействии с заинтересованными сторонами и бенефициарами, а их качество нелегко оценить. В данной статье предложен инструмент оценки политики, разработанный для постановки ряда вопросов о влиянии политики и вмешательств на справедливость в сфере здравоохранения, ответы на которые могут быть получены на основе эмпирических данных или принципов общественного здравоохранения. Результаты могут оцениваться по силе воздействия и направлению вероятных эффектов и могут дать краткое представление о том, как политика или инициатива способна увеличить или уменьшить неравенство в сфере здравоохранения. Эти вопросы могут использоваться как часть аналитического обзора для оценки воздействия. В статье приведено описание применения этого подхода к оценке трех мер политики, ориентированных на население, для определения их возможного влияния на неравенство в отношении ожирения: (i) меры по ограничению воздействия на детей рекламного маркетинга вредных продуктов и напитков; (ii) меры по изменению состава продуктов питания и напитков; (iii) меры по улучшению закупок продуктов питания для государственных учреждений.

## Introduction

Diet-related chronic diseases, including obesity, account for more than a quarter of premature deaths from noncommunicable diseases worldwide, and the large majority of these deaths occur in low- and middle-income countries.^1^ Policies to improve population dietary patterns and reduce obesity prevalence can help nations meet their targets in the sustainable development goals for reduction of noncommunicable diseases. The World Health Organization (WHO) acceleration plan to stop obesity emphasizes the need for policies that recognize societal rather than individual responsibility, and argues for “multisectoral actions that can have a more direct impact on the disease (such as food manufacturing, marketing and pricing) and others that seek to address the wider determinants of health (such as poverty reduction and urban planning).”^2^

Both the WHO acceleration plan and its accompanying delivery framework^3^ note the existence of disparities in health status between and within populations, but neither document addresses this issue directly. Policy-makers are aware that disparities exist in the prevalence and risk of obesity and most noncommunicable diseases between populations of different social and economic status, including migrant status, urban–rural residence, sex, ethnicity, educational status and household wealth. Furthermore, such disparities may be inequitable and reflect a failure of governments to protect the “right of everyone to the enjoyment of the highest attainable standard of physical and mental health” under the International Covenant on Economic, Social and Cultural Rights.^4^ Such inequities are also economically harmful as they can impose unnecessary personal and societal costs through ill-health, reduced educational attainment, lower productivity in employment and diversion of family resources to providing care. Consequently, for both legal and economic reasons as well as ethical concerns, policy-makers try to avoid creating or increasing health inequities.

Various tools have been published which are designed to assist policy-making bodies in their consideration of policy options, based on the general approach known as health impact assessment. These tools provide procedures to screen policies, scope their impact and mitigate or monitor their health outcomes,^5^ and some guidance is available for policy-makers.^6^^–^^8^ The tools may be supplemented with health equity information about population disparities and health gradients to consider disparities in the health impact assessment process. This process is well designed to assess how a policy might be implemented effectively and acceptably, but is not so well designed to assess in advance the likely equity effects, and to do so based on available evidence. This drawback may be one of the reasons why many national nutrition policies to tackle obesity fail to include actions specifically designed to improve health equity.^9^

In 2020–2023, the European Union Joint Action Best-ReMaP (Best practices in Reformulation, Marketing and Public procurement)^10^ brought together 36 government agencies and research bodies concerned with obesity and public health nutrition to assess best policies and practices. The focus was on three approaches: (i) policies to restrict children’s exposure to the promotional marketing of unhealthy foods and beverages; (ii) policies and interventions for food and beverage reformulation; and (iii) policies and interventions to improve food procurement for public institutions (especially schools). During the course of the Best-ReMaP Joint Action, a recurring question asked by government officials was whether it was possible to determine in advance if a proposed policy or initiative was likely to widen or narrow social disparities in nutrition or obesity status.

To answer this question, the Best-ReMaP Joint Action considered whether a preparatory equity risk assessment phase could be conducted as a desk-based exercise to support policy-makers who needed an evaluation tool before any participatory consultation. Such a tool would use available evidence on pre-existing risks and vulnerabilities, responsiveness and acceptability, along with recognized public health principles, to anticipate the likely equity effects.

## Risk assessment approach

Classical risk assessments are undertaken to prevent avoidable exposure to hazardous environments. These assessments include the identification of hazards, persons who might be harmed by the hazards, and the severity and likelihood of the risk. Further consideration may be given to different vulnerability to harm and preparedness to avoid harm; individual self-efficacy, knowledge and motivation;^11^ and community support and other forms of social capital which may reduce harm.^12^^,^^13^

As part of the Best-ReMaP Joint Action, the so-called hazard and harm approach was adopted, and a rapid literature review was done focused on obesogenic environments.^14^ Consideration was given to what forms of evidence could help show if an intervention to change the environment (a change in a hazard) might cause an increase or decrease in health status disparities (an increase or decrease in the harm). For example, what evidence would help to determine if a restriction on promotional marketing of less heathy foods to children might influence health-related behaviour or body weight? The Best-ReMap Joint Action review was published as grey literature in the project results,^15^ and the conclusions about the types of evidence needed to make assessments of the impact on health equity are summarized in Box 1. It should be noted that the original review included more sources than reported here, and that the box now includes some material published after the original review.

Box 1 **Examples of framing equity impact and evidence needs**

***General equity assessment* *^6^* ^,^ ^7^ ^,^ ^16^**

- Current health disparities (e.g. relating to socioeconomic status, residence, ethnicity and sex).
- Disparities in health behaviours (e.g. dietary patterns).
- Disparities in exposures to hazard (e.g. targeted marketing).
- Disparities in sensitivity to hazards (e.g. response to marketing and response to food labelling).

***Effectiveness of intervention for different population groups (e.g. literacy, numeracy and financial barriers)***

- Disparities in protective services (e.g. clinical screening and health promotion services).
- Disparities in barriers to protective services (e.g. accessibility and discrimination).

***WHO and Health In All Policies frameworks* *^17^* ^–^ ^19^**

- Different exposure to risk (e.g. affordability (prices of vegetables, fruit compared with prices of energy-dense, low-nutrient, processed food), and exposure to promotional marketing of foods and fast food brands).
- Different neighbourhoods (e.g. availability of healthier products, exposure to unhealthy food outlets, exposure to neighbourhood advertising of unhealthy foods, and access to beneficial food services such as pre-school, school and care home).
- Different vulnerability (e.g. acceptability (dietary exclusions and requirements, and fasting periods), ability to read food labels or nutrition information, and skills and equipment to prepare nutritious foods).
- Different access to support (e.g. distance and cost barriers to accessing health-service consultations (child growth check-ups), language and belief barriers to accessing health advice, discrimination or different treatment, and comprehension of advice).
- Different effects of consequences (e.g. increased need for long-term support services, need for household resources, impact on self-esteem and social inclusion and/or exclusion, and economic effects of ill-health including lost productivity and premature death).

***RE-AIM framework* ^20^ ^–^ ^23^**

- Reach. Can the intervention access those in greatest need and all members of those subgroups? These groups include hard-to-reach people such as homeless people excluded from household survey data.
- Effectiveness (or efficacy). Are there disparities in effectiveness, or in the measurement of effectiveness?
- Adoption. Are there disparities in the likelihood of adoption of behaviour change, and disparities in barriers to policy implementation (e.g. in the ability of commercial operators to undermine behaviour change)?
- Implementation. Are there disparities in completeness of an intervention or the ability to afford the financial or time costs implied by an intervention?
- Maintenance. Are there disparities in the ability of people to maintain their change in health behaviour?

***Additional public health frameworks* *^8^* ^,^ ^24^ ^–^ ^32^**

- Micro versus macro. Interventions are categorized as being implemented at the local, targeted level (micro), in the wider community or at the national level (macro). Evidence suggests health inequities are more likely to be reduced with macro-level policies.^24^
- Racial, cultural and ethnic dimensions. Interventions are assessed on their relevance to and likely impact on pre-existing disparities and discriminatory practices.^8^
- Agentic versus structural. Interventions are assessed on a spectrum from agentic (interventions that rely on behaviour led by individual choice) through mixed agentic and structural interventions, to purely structural ones (interventions that alter the social, economic, political and material context in which a behaviour occurs).^25^^–^^28^
- Nuffield ladder of interventions. This dimension spans interventions that are most likely to increase health inequalities to those that are least likely. The ladder starts with providing information at the individual level, enabling choice at the household level, guiding choice at the local level, restricting choice at least at the community level, and eliminating choice at the societal level (e.g. smoking ban applied to all).^29^
- Universal and proportionate. This dimension emphasizes the importance of a combination of population-wide and targeted interventions that are, ideally, simultaneously universal (i.e. are applied across the population in need) and proportionate (i.e. have an effect in proportion to need).^30^
- Threats and weakness analyses. These analyses examine the likelihood of challenges to structural support and empowerment, including social barriers (e.g. discrimination) and commercial threats (e.g. promotion of unhealthy products and legal challenges to equity-promoting policies).^31^^,^^32^

RE-AIM: reach, effectiveness, adoption, implementation and maintenance; WHO: World Health Organization.

## Framing the assessment

Box 1 provides examples of the types of evidence that can support an equity risk assessment, starting with the general empirical assessment of exposure, vulnerability and responsiveness, and complemented by types of framework to help structure the assessment. For example, the framework used by the Commission on the Social Determinants of Health^30^ emphasizes the importance of a combination of population-wide and targeted interventions that are, ideally, both universal (i.e. are applied across the population in need) and proportionate (i.e. have an effect in proportion to need). This approach is also reflected in other frameworks, which consider upstream or population-wide approaches, contrasted with downstream or localized or individualized approaches, and also systems-wide, structural interventions in contrast to person- or family-targeted (agentic) interventions.

Research has shown that population-wide, structural interventions have been frequently absent from obesity-reduction policies, while agentic interventions are common.^9^ Interventions which depend on individual agency to improve health behaviour typically rely on information and education to increase knowledge, skills or empowerment of individuals to make healthier choices. Interventions at the structural end of the spectrum typically reduce or remove individual agency, making the healthier option the main or only option in a given context. Such interventions are typically enacted through standards-setting or regulatory changes. The agent–structural framing can help to identify the types of intervention that require an individual, family or community to supply resources which are unevenly distributed in society (e.g. financial resources, time, skills, and numeracy and literacy including health literacy). Interventions may also be agent–structural if they alter structural conditions to make health-enhancing behaviours more appealing, accessible or affordable, while also ensuring individuals still have the right to make the same health behaviour choices that were available before the intervention. Evidence suggests health inequities are more likely to be reduced with structural or agent–structural interventions,^33^ especially if implemented across several settings.^34^

From the sources indicated in Box 1, we distilled four specific criteria that could be used to make assessments of the health equity effects of a policy or intervention. The four criteria are: (i) pre-existing exposure to risk, including pre-existing exposure to a hazard, health disparity and susceptibility to a hazard; (ii) exposure to the policy or intervention, that is, reach and type of an intervention, including reach across and penetration into all subgroups, localization, population-wide or targeted, and availability of and access to supportive measures; (iii) response to a policy or intervention, including agency-led or structure-led behaviour change, requirements for skills and resources, transfer between settings, and cultural acceptability and priority; and (iv) response sustainability, including community compatibility, community and regulatory support, presence of barriers and exposure to threats to implementation.

## Operationalizing the assessment

In the Best-ReMaP project, the criteria identified previously were discussed and the project participants requested more concrete examples of how the assessment would work in practice. The four criteria provided a conceptual framework which led the participants to ask for a set of questions or a checklist that could be applied to a proposed policy or intervention. Such questions would need to be operationally clear, with suggested forms of evidence that would indicate the direction of the effect – that is, increasing or decreasing health inequity – and where possible allowing the user to qualify the strength of the evidence or to note its absence.

Table 1 shows examples of the types of operational questions that would be needed to make an assessment based on the four framework criteria. The table is based on the three policy areas in the Best-ReMaP analysis, namely: restricting children’s exposure to marketing of foods high in fats, sugars or salt; promoting reformulation of foods; and using public procurement policies to promote healthier diets. Other types of policy or intervention – such as taxing sugar-sweetened beverages, offering community weight management classes, providing fruit and vegetable vouchers, or restricting fast food outlets near schools – might need their own operational questions within the framework criteria.

**Table 1:** Operationalizing health equity risk assessment: approach of the policy assessment tool for health equity

Domain for assessing inequities	Assessment criteria	Evidence needed for Best-ReMap policy areas	Sources of evidence
Pre-existing risk: evidence of underlying disparities in the need for an intervention	Historical risk: pre-existing exposure shown in health disparities	Are there disparities in diet and nutrition status (e.g. sugary drink consumption and overweight prevalence)? Who would benefit most from change?	Survey data from local and national populations, and from comparable populations elsewhere Scientific studies and reviews Data from social service departments and education departments Businesses including market data, sales data and client usage or uptake data Surveys and reports by non-profit consumer and health organizations
	Current risk: general and targeted exposure to potential hazard	Are there disparities in exposure to hazards? For example, are some subgroups more exposed to television commercials or targeted as consumers, and are school meals subsidized for some groups?	
	Variation in vulnerability or susceptibility	Are there subgroups that are more susceptible to a hazard (e.g.to susceptible to advertising messages, price modification or product health claims)?	
Reach and type of policy or intervention: evidence of the universality of an intervention and the likelihood of (and barriers to) reaching the groups most needing the intervention	Reach across subgroups and across a socioeconomic gradient	Do public health initiatives reach all groups? Are there language issues? For example, does advertising restriction reach all groups? Do health promotion messages reach all, especially those in greatest need?	Reports and assessments of prior health education engagements Evidence of language barriers and other cultural barriers Businesses including survey data to identify market distribution and penetration in subgroups Scientific studies and reviews Surveys and reports by non-profit consumer and health organizations
	Degree of penetration within subgroups	Within poorer groups, will an intervention reach all participants? For example, are homeless families and non-native language speakers reached?	
	Localized (micro) or widespread (macro)	Is the policy local or national? For example, do advertising restrictions reach all those exposed? Are reformulated foods available and accessible to all households?	
	Upstream or downstream	Does the policy target upstream (e.g. food manufacturers and food advertisers) or downstream (e.g. shoppers, families and children)? Whose behaviour is being targeted?	
	Reach of supportive messaging	Do social marketing campaigns supporting policies reach the people most affected? For example, do health messages in support of reformulation regulations reach all consumers?	
	Access to supportive services	Do supporting services reach the people most affected? For example, do school meals vouchers reach all those in need? Are price subsidies passed on to all consumers?	
Response to an intervention: evidence on the anticipated acceptability and feasibility of an intervention across all groups and for those with greatest need	Agency- or structure-led behaviour change	Does the policy require individual voluntary changes in behaviour? For example, are reformulated foods the easiest choice? Do parents have to monitor television advertising schedules?	Responses to previous health improvement programmes; for example, evidence that policy implementation requires individuals to make voluntary behaviour change or use of their own resources (including financial and time resources). Data from relevant community organizations (e.g. faith groups, worker groups and mothers’ networks)^a^
	Resource requirements	Does behaviour change require financial, time or equipment resources? For example, are reformulated products more expensive? Are school-meal vouchers sufficiently valuable to attract regular use?	
	Skills, literacy and numeracy requirements	Does behaviour change require skills to implement? For example, do food labels need literacy or numeracy skills? Do heath claims require nutrition competency? How are school-meal vouchers obtained?	
	Transfer of behaviour changes	Do health behaviour improvements transfer between settings? For example, do school-based fruit promotion programmes affect home-based dietary patterns?	
	Household acceptability of an intervention	Are behaviour changes suitable for all cultures? For example, are school-meal vouchers acceptable for halal, kosher or specific medical dietary needs?	
	Household perceived priority of an intervention	Is the behaviour change competing with other priorities? Are all members of a household motivated? For example, is there evidence that parents support television advertising restrictions and make use of meal vouchers?	
Sustainability of response: evidence on the longer-term maintenance of an intervention across the population in need and in proportion to need	Compatibility with community and cultural environment	Do policies concur with existing dietary patterns and food supply environments, especially for culturally specific foods?	Local and national population surveys and scientific literature Data from relevant community organizations^a^
	Voluntary or regulatory	Does the policy have statutory support? Can it be ignored in some areas, or reversed easily? For example, is reformulation a regulatory requirement or a voluntary code of practice?	
	Barriers and threats to policy maintenance	What might undermine the policy? For example, can fast food outlets operate close to school premises, with special offers at lunch-time? Are reformulated foods only available in larger stores? Do the barriers or bottlenecks affect some communities or subgroups more than others?	

^a^ This evidence can be considered as a preparatory scoping stage for a subsequent health impact assessment.

Note: examples are drawn from the three Best-ReMaP policy proposals.^10^

## Evidence requirements

The evidence required to assist an operational evaluation of the impact on health equity may vary in its availability, reliability and quality. For the purposes of a rapid assessment, the Best-ReMaP project graded the evidence into five categories: strong evidence in favour of improving health equity; moderate evidence in favour of improving health equity; moderate evidence for worsening health equity; strong evidence for worsening health equity; and lack of evidence either way.

The evidence ratings can be supplemented with notes on the certainty of each assessment, and a weighting system could be developed if considered necessary. An overview can be taken within each of the four criteria in the framework and a summary of the overall likely outcome can be produced. The experience of the Best-ReMaP participants suggests that a simple favourable or unfavourable summary is enough for internal purposes, but that a more detailed description might be needed if a proposed policy was likely to be challenged.

For the Best-ReMaP project, the framework was used to provide a one-sheet summary for each of the three policy areas: policies to restrict children’s exposure to the promotional marketing of unhealthy foods and beverages;^35^ policies and interventions for food and beverage reformulation;^36^ and policies and interventions to improve food procurement for public institutions (especially schools).^37^ The summary sheets also provide a grading of the evidence, with colour coding to assist rapid assessment. These sheets have been published on the project website, along with a more detailed analysis, citing data sources and evidence assessment.^38^

## Discussion

The results described in this paper were presented and discussed at the final conference of the Best-ReMaP Joint Action, and publication of the work was encouraged by the steering group for dissemination to persons responsible for policy formulation.

Several limitations and qualifications were noted. First, the assessment process for the quality of the work was spread over several online meetings of researchers and government officials, supported by offline consultation which allowed more considered responses but less opportunity for interactive discussion. The work presented in our paper should therefore be considered a prototype version of a risk assessment framework.

Second, the Best-ReMaP Joint Action concerned the policy environment common to European Union member states and this raises questions about its specificity to the region and its transferability. Extending the framework to other policy environments is expected to likely require some adaptation of the assessment criteria. For example, a 2024 paper^8^ provides a perspective from North America, with a greater focus on community action, monitoring and attention to the voices of those people affected by policies – all of which are core parts of the process of a health impact analysis. 

Beyond these specific limitations, a further problem was the substantial gaps in empirical evidence on disparities in the impact of a policy. In an umbrella review of food environment policies with the potential to reduce inequalities in diet, it is suggested that the strongest evidence is for food taxation, but that “for all other policy areas the evidence base was poor.”^39^ A systematic review of three policy areas – marketing to children, nutritional front-of-pack labelling and food taxation – found that both marketing and taxation policies were likely to reduce inequities in obesity risk for children, but that front-of-pack labelling was less likely to reduce inequities (except if it led to product reformulation). In all cases the evidence was weak.^40^ Similarly, a 2014 study concluded that structural interventions were likely to reduce inequities, but the evidence was weak.^26^

The evidence for some of the indicators of exposure to hazards and vulnerability to their effects may be available through survey literature (including market surveys), while data on reach and penetration of interventions may not be available. The scoping for a health impact assessment may look at these areas and not reach a conclusion on the potential disparities of the impact on health. Our paper suggests a more systematic approach that allows a wider range of evidence to be used for assessing the likely impact of a policy on health disparities.

## Conclusion

The equity assessment framework presented in this paper is proposed as a tool (policy assessment tool for health equity (PATHE)) to assist in policy development, taking nutrition policies and obesity as a case study. The tool was developed in response to a request for a structured format to help government officials to make an advance assessment of the likelihood that a proposed policy or initiative would widen or narrow inequities in nutrition status. Our paper proposes a risk assessment framework to evaluate health inequities, and provide indications of the effect of proposed interventions or policies on widening or narrowing such inequities. The framework has four elements and may be implemented with a series of questions that can be answered using available empirical evidence and public health principles.

The evidence needed can be sought in a range of sources including surveys, empirical studies, reports of community organizations and public health nutrition principles. The tool is designed to provide a preparatory assessment of the impact of a proposed policy, and can support health impact assessment scoping, consultation, monitoring and review stages. The tool can also be used to precede or complement specific equity-focused discussions in the consultation process of a health impact assessment, for example the suggested equity-focused health impact assessment,^25^^,^^26^ and can provide a briefing for participants in this assessment.

Many gaps still exist in the evidence on which policies can be assessed. Our tool can help make preliminary judgements, but stronger evidence is still needed. We urge researchers and research-funding bodies to ensure that the reporting of outcomes of interventions include participants’ socioeconomic status. We also urge WHO to help strengthen the evidence on the impact of policies on health as a means of supporting Member States seeking to introduce effective and equitable policies.
